# Group treatment for complex dissociative disorders: a randomized clinical trial

**DOI:** 10.1186/s12888-022-03970-8

**Published:** 2022-05-16

**Authors:** Harald Bækkelund, Pål Ulvenes, Suzette Boon-Langelaan, Espen Ajo Arnevik

**Affiliations:** 1 Research Institute, Modum Bad Psychiatric Hospital, Vikersund, Norway; 2 Section for Implementation and Treatment Research, Norwegian Center for Violence and Traumatic Stress Studies, Oslo, Norway; 3 Department of Psychology, Faculty of Social Sciences, University of Oslo, Oslo, Norway; 4 Private practice, Maarssen, The Netherlands; 5 Section for clinical addiction research, Oslo University Hospital, Oslo, Norway

**Keywords:** Dissociative disorders, Randomized clinical trial, Group psychotherapy, Psychological trauma, Psychotherapy outcome research

## Abstract

**Background:**

Patients with complex dissociative disorders (CDD) report high levels of childhood- abuse experiences, clinical comorbidity, functional impairment, and treatment utilization. Although a few naturalistic studies indicate that these patients can benefit from psychotherapy, no randomized controlled trials have been reported with this patient-group. The current study evaluates a structured protocolled group treatment delivered in a naturalistic clinical setting to patients with CDD, as an add-on to individual treatment.

**Methods:**

Fifty nine patients with CDD were randomized to 20 sessions of stabilizing group–treatment, conjoint with individual therapy, or individual therapy alone, in a delayed-treatment design. The treatment was based on the manual *Coping with Trauma-Related Dissociation*. The primary outcome was Global Assessment of Functioning (GAF), while secondary outcomes were PTSD and dissociative symptoms, general psychopathology, and interpersonal difficulties.

**Results:**

Mixed effect models showed no condition x time interaction during the delayed treatment period, indicating no immediate differences between conditions in the primary outcome. Similar results were observed for secondary outcomes. Within-group effects were non-significant in both conditions from baseline to end of treatment, but significant improvements in psychosocial function, PTSD symptoms, and general psychopathology were observed over a 6-months follow-up period.

**Conclusion:**

In the first randomized controlled trial for the treatment of complex dissociative disorders, stabilizing group treatment did not produce immediate superior outcomes. Treatment was shown to be associated with improvements in psychological functioning.

**Trial registration:**

Clinical Trials (NCT02450617).

**Supplementary Information:**

The online version contains supplementary material available at 10.1186/s12888-022-03970-8.

Dissociative Identity Disorder (DID) is the most severe of the dissociative disorders described in the fifth edition of the Diagnostic and Statistical Manual of Mental Disorders [1]. DID is characterized by having different dissociative identities, often referred to as parts or selves, with reported disruptions in memories, sense of self, and agency. Patients report dissociative amnesia, involving a lack of recall of autobiographical material both in daily-life and for traumatic events. DID and the closely related Other Specified Dissociative Disorders, example 1 (OSDD), where similar disturbances are observed without meeting the full clinical picture of DID, are commonly categorized as Complex Dissociative Disorders (CDD) [2]. CDDs are among the most costly psychiatric conditions, both in terms of societal resources and individual suffering [3]. Patients with CDD suffer from severe psychiatric symptoms, high levels of comorbidity with other disorders, low level of psychosocial functioning, and frequent suicidality and self-destructive behavior [4–8]. Studies indicate that the prevalence of DID in psychiatric populations might be as high as 5% [9].

CDDs are associated with early traumatization and patients predominantly report being victims of childhood abuse, especially sexual abuse [4, 10]. Researchers and clinicians commonly view dissociation as a response to severe trauma that allows the individual to cope with distress in the immediate aftermath, but may lead to the development of dissociative disorders later [10]. The personality disruptions and amnesia characteristic of DID are generally understood as the individual’s effort to defend against or *compartmentalize* memories, feelings, and sensations related to trauma [11]. An overarching goal of treatment is to gradually overcome this compartmentalization between dissociated parts of the personality and achieve a more integrated identity and functioning [12].

Some scholars contend however that factors such as fantasy proneness and suggestibility, cultural expressions (films, books, internet fora, etc.), or suggestive therapeutic practices, cause dissociation and dissociative disorders, not trauma. CDDs are in this perspective not viewed as genuine psychiatric conditions, but as a result of *iatrogenic* therapeutic mistreatment and cultural superstitions. Accordingly, it has been proposed that treatment of CDDs may be potentially harmful, since treatment may reinforce beliefs about multiple personalities and trauma through suggestive influences [13–15], although no empirical evidence has been presented that support this assertion [16].

At present, no evidence-based guidelines for the treatment of DID exist and very few clinical studies on this patient group have been published. Practice-based guidelines developed by the International Society for the Study of Trauma and Dissociation (ISSTD) [12] recommend a phased-based approach, with three stages. The first *stabilization* phase is focused on establishing safety, increasing control over symptoms, and improving psychosocial functioning. Skills training is recommended as an essential part of this treatment-phase, to facilitate the patients’ ability to increase safety, regulate emotions, tolerate distress, and improve interpersonal functioning. When the patient is sufficiently stabilized, treatment may progress to the second phase where traumatic memories are confronted and processed. The final third phase of treatment addresses rehabilitation and reintegration of personality states. Throughout treatment, the guidelines recommend interventions that explicitly address dissociation, including identifying and addressing different self-states or parts of the personality [12]. This is thought to help the patient to improve inner communication and cooperation between parts of the personality, and thereby improve control and functioning. Although individual therapy is recommended as the primary treatment modality, the guidelines recommend group treatment as a valuable adjunct treatment to facilitate skills training and counter a sense of isolation. Groups are recommended to be time-limited, highly structured, and focused [12].

A review from 2009 identified eight studies on the treatment of dissociative disorders, all non-randomized and without a control group [17]. Overall, treatment was associated with a reduction in both dissociative and other symptoms from pre- to post-treatment, with medium to large effect sizes. A later naturalistic study of inpatient treatment of patients with sexual abuse histories [18], reported moderate effects of treatment on PTSD symptoms and general symptomatology in 24 patients with CDD from assessment to follow-up. However, changes in dissociative symptoms were insignificant and treatment gains were generally lower than in patients without CDD. Also, Brand and colleagues [19–21] followed the outpatient treatment of 226 patients with CDD, recruited through their therapist, in a longitudinal observational study. Treatment was associated with a decrease in symptomatic distress (dissociation, PTSD symptoms, and general psychopathology), reduction of self-destructive behavior (self-harm, substance abuse, and hospitalizations), and improvement in psychosocial functioning. These gains were maintained at a six-year follow-up and associated with economic cost-savings [21, 22]. However, the participating patients had been in treatment for an average of 5 years with their current therapist, and a substantial number were still in treatment 6 years later, showing the substantial therapeutic effort required to achieve these gains. Also, treatment in this study was not protocolled, although a majority of therapists proclaimed to use interventions recommended by ISSTD guidelines [23]. The only clinical study to date using a standardized intervention investigated the safety and effects of a web-based education program focused on stability and safety [24]. In an internationally recruited sample of 111 patients with CDD, the online intervention completed together with regular individual therapy was associated with decreases in dissociation, PTSD symptoms, and self-destructive behavior, while emotion regulation and adaptive capacities improved. Treatment gains increased over 2 years and were larger in patients with high initial dissociation scores. As with all previous studies though there was no control group and since patients were requited through interested therapists the generalizability of the results might be limited by selection bias.

Although previous studies indicate that patients’ CDD can improve while receiving psychotherapy, there is a clear need for more methodologically rigorous studies to make more solid inferences about treatment. Most notably, randomized allocation and inclusion of a control condition make causal inferences about treatment efficacy possible. Structured and protocolled treatments, based on specific psychological theories, will allow replication and inform the therapist about interventions associated with progress. Also, participant inclusion should be less restricted and based on normal clinical requirements, to decrease selection bias and ensure generalizability to ordinary clinical settings.

The current study therefore aims to evaluate a structured protocolled group treatment delivered in a naturalistic clinical setting to patients with CDD, as an add-on to individual treatment. Following ISSTD guidelines, the group program is time-limited, highly structured, and focused on skills training, to facilitate the goals of the first phase of stabilizing treatment. The study also included a control condition of ordinary individual treatment in a delayed treatment design. We predicted that participation in group treatment combined with individual treatment would lead to greater improvements in psychosocial function and reduction of psychiatric symptoms, compared to individual treatment alone.

## Materials and methods

### Setting and participants

**Fig. 1:**
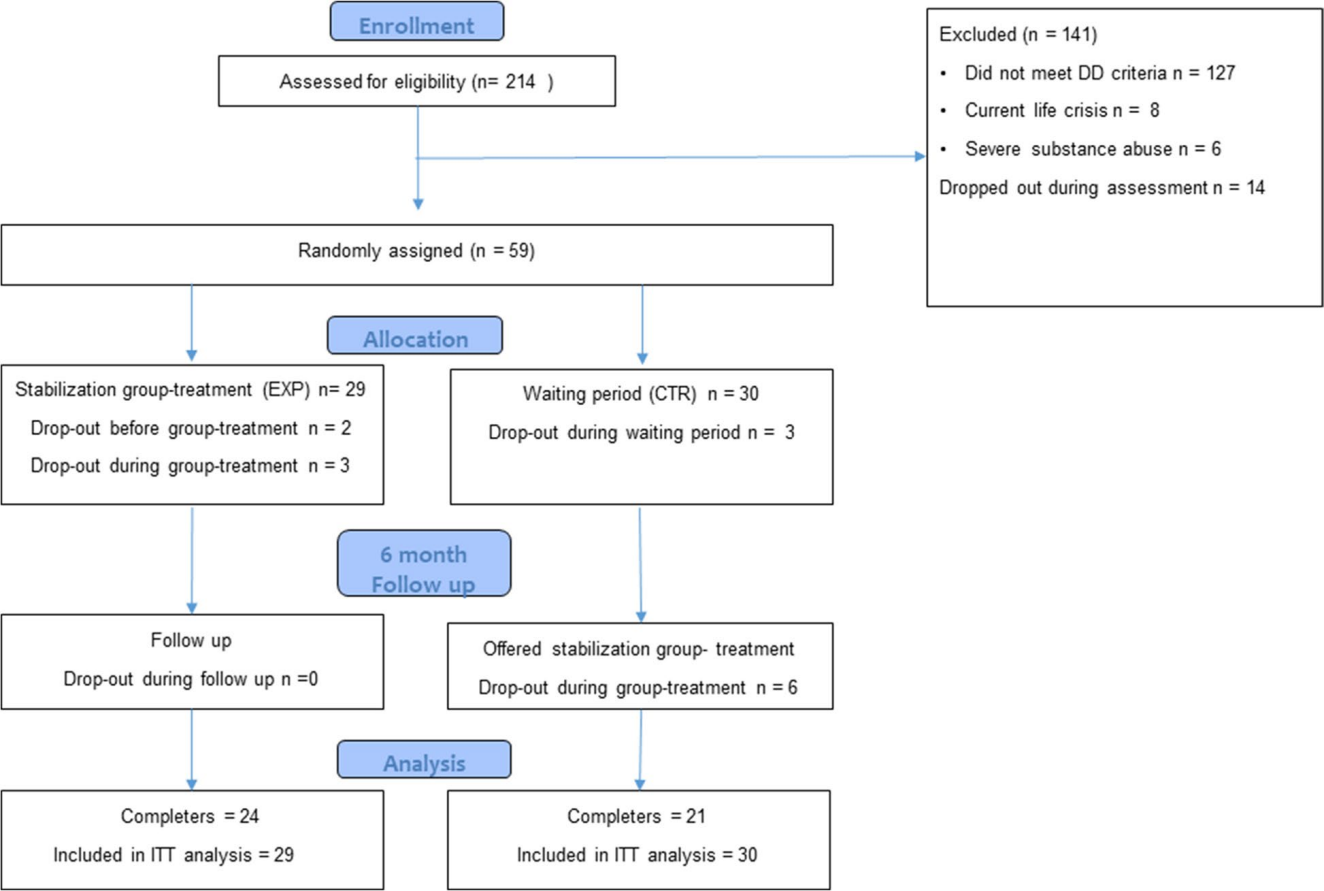
Flow-diagram of a randomized controlled trial of group treatment for complex dissociative disorders

The current study was conducted at an outpatient clinic that accepts referrals of patients with reported trauma histories and trauma-related difficulties. The clinic primarily offers specialized group treatment, and patients are required to have planned or ongoing individual treatment at another clinic or private practice to be admitted. Recruitment of patients started in September 2014 and ended in November 2017. All referred patients were invited to participate at intake and written informed consent was obtained. Further inclusion or exclusion was based on the following structured diagnostic interviews described later. Inclusion and exclusion criteria were the same as those used by the clinics’ treatment program for dissociative disorders. All included patients had to: (1) meet the criteria for a diagnosis of Dissociative Identity Disorder or Other Specified Dissociative Disorders, example 1 according to DSM-5 criteria [1]; (2) be between 18 and 65 years of age; (3) have sufficient competence in Norwegian to be able to participate in a psychoeducational group. Exclusion criteria were: (1) acute suicidality; (2) severe substance abuse interfering with treatment; (3) ongoing psychotic episode; (4) current life – crisis interfering with therapy (e.g. ongoing abuse, divorce, court case, a somatic disease in spouse or children, etc.); (5) neurological disease, mental disability or life-threatening somatic disease. More information about recruitment and patient-flow is outlined in Fig. 1.

The study was funded by Modum Bad Psychiatric Hospital. All procedures comply with the ethical standards of the relevant national and institutional committees on human experimentation and with the Helsinki Declaration of 1975, as revised in 2008. The study was approved by the Norwegian Regional Committees for Medical and Health Research Ethics (2013/2350).

### Design and randomization

**Fig. 2:**
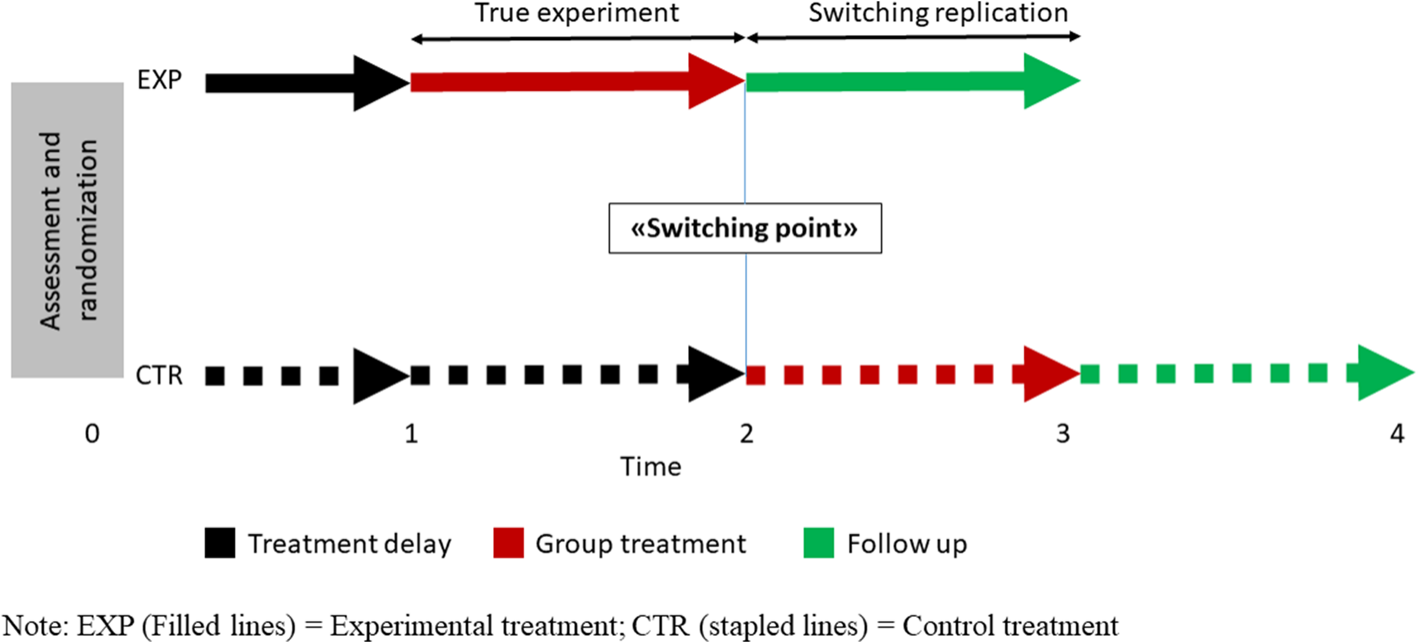
Illustration of study design combining a randomized trial with a delayed treatment control group and multiple time series with switching replication

As previously described this study recruited participants from referred patients to an ordinary clinical service, and a no-treatment control group was therefore difficult and unethical to employ. We therefore chose a delayed-treatment design, where included patients were randomized to either the group intervention immediately (EXP) or after a corresponding waiting period (CTR). All participants continued with their conventional individual treatment throughout the study period, including the waiting period for the CTR group. With this design, all patients receive the intervention, and the mean waiting time from ordinary clinical practice was not prolonged. A delayed-treatment design also allows for both a “true experiment” comparing the intervention with a control group and a switching replication when the control group later receives the intervention (Fig. 2). The “switching point”, between these two comparisons, was the primary outcome assessment. To investigate long-term effects we also assessed participants 6 months after the end of the intervention.

Following assessment and inclusion, participants were randomized to conditions by an independent administrative assistant not involved in the research group. Random sequences were generated at www.graphpad.com. Researchers and assessors involved in the study were not informed about condition assignment, to ensure blind assessment of outcome measures. The protocol originally planned to include 72 participants (36 in each condition), based on a priori power analysis. However, due to a lower prevalence of CDD in the study population than predicted and resource constraints, the final sample recruited was 59 participants. Other deviations from the protocol were minor changes in the treatment program and changes in secondary outcome measures (see supplementary). The trial was preregistered on May 21th 2015, at Clinical Trials (NCT02450617).

### Treatment

The experimental treatment studied consisted of twenty 90-minutes sessions of psychoeducational group therapy, offered adjunct to the participants’ conventional individual treatment. The treatment was based on the published manual *Coping with Trauma-Related Dissociation* [25]. This manual relies on a theoretical understanding of dissociation and dissociative disorders rooted in the theory of structural dissociation [26, 27], defining dissociation as a core feature of trauma that involves a division of the personality into distinct dissociative parts. This division manifests in the severe psychoform and somatoform dissociative symptoms in CDD. The treatment teaches an understanding of different dissociative parts and skills to facilitate *inner cooperation* between parts. The treatment also draws upon different therapeutic orientations such as cognitive-behavioral therapy, mindfulness, and short-term dynamic therapy. The focus of the manual is on the first stabilization phase of treatment [12]. Each of the 43 chapters consists of educational pieces to foster the participants understanding of their disorder and instructions for coping-skills and homework. The experimental treatment in the current study was reduced to 20 sessions since it was infeasible to delay treatment for the control condition for 43 weeks. Also, clinical experience at the clinic indicated that many patients profited from a shorter psychoeducational program for early stabilization, before moving on to more individual work or different group programs. The 20 topics were selected by the first author of the treatment manual, based on clinical experience and rationale to provide the best early stabilization. The themes of these chapters can be seen in Table 1. As outlined by the manuals *guide for trainers*, each group was led by two therapists and had nine participants. To enhance each participant’s feeling of safety and predictability all sessions were highly structured, with psychoeducation, structured group discussion, and skills exercises. Each session included guided exercises in skills and techniques to handle symptoms, such as grounding exercises and “safe place”, and homework focused on using these skills. In addition, participants had access to written material and video recordings made by the therapist repeating the educational pieces and exercise instructions of each session. These could be used by participants to repeat the content when they wanted.

**Table 1:** Psychoeducational topics in a group program for complex dissociative disorders, based on *Coping with Trauma-Related Dissociation* (Boon et al., [25])

Session	Topic	Chapter in manual
1	Introduction. Rules, regulations, and motivation	
2	Learning to regulate yourself (1)	18
3	Understanding dissociation	1
4	Symptoms of dissociation	2
5	Understanding dissociative parts of the personality (1)	3
6	Understanding dissociative parts of the personality (2)	3
7	Overcoming phobia of inner experience	5
8	Learning to reflect	6
9	Beginning to work with dissociative parts	7
10	Developing an inner sense of safety	8
11	Summary and review of content so far	
12	Establishing a healthy daily structure	10
13	Improving sleep	9
14	Understanding traumatic memories and triggers	14
15	Coping with triggers (1)	15
16	Learning to regulate yourself (2)	18
17	Inner cooperation	27
18	Planning for difficult times	16
19	Preparing for saying goodbye	35
20	Evaluation and leave-taking	

The conventional individual treatment of each patient was not protocolled or experimentally manipulated, but delivered as seen fit by the patients’ individual therapists throughout the study period. Individual therapists were psychologists, psychiatrists, or nurses working in other clinical departments or private practice (see supplementary). All individual therapists were informed about the results of the diagnostic assessment. They were invited to a meeting at the start of the group and informed about the rationale and content of the intervention. While in group treatment, patients were encouraged to discuss their experiences from the group, discuss homework, and share written material with their individual therapists.

### Measures

#### Diagnostic assessment

*The Structured Clinical Interview for DSM-IV Dissociative Disorders* [28] was used for the diagnostic evaluation of dissociative disorders. SCID-D assesses five dimensions of dissociative symptoms (amnesia, depersonalization, derealization, identity confusion, and identity alteration) on a four-point scale (1 = ‘none’, 2 = ‘mild’, 3 = ‘moderate’, 4 = ‘severe’), based on the patient’s descriptions and observations during the interview. The information and scoring obtained during the interview were used to diagnose patients based on DSM-5 criteria. All diagnostic evaluations were done jointly by clinicians with extensive training and experience in diagnosing dissociative disorders.

*The Multidimensional Inventory of Dissociation* [29] is an extensive self-report measure of pathological dissociation. It consists of 218 items scored on an 11-point Likert scale. Of these, 168 items measure different experiences of dissociation and 50 measure validity. In the present study, MID scores were used primarily as supplementary information toward a diagnostic decision on CDD status.

*The Post-traumatic Symptom Scale – Interview (PSS-I)* [30] was used to diagnose PTSD according to DSM-5 criteria. PSS-I has been shown to have good interrater reliability and convergent validity with other PTSD measures [31].

*The Mini-International Neuropsychiatric Interview, version 6.0* [32] was used to assess general psychopathology. The Norwegian translation has been shown to have acceptable psychometric properties [33].

*Childhood Trauma Questionnaire – Short form (CTQ-SF)* [34] was administered to assess self-reported history of childhood trauma and abuse. The 28 items of CTQ-SF are scored from 0 (“never true”) to 5 (“very often true”). The measure has five subscales: Emotional neglect, physical neglect, emotional abuse, sexual abuse, and physical abuse. The Norwegian translation of CTQ-SF has shown good psychometric properties [35].

To record background information and sociodemographic data we used a generic form.

### Primary outcome

*Global Assessment of Functioning – Split version (GAF-S)* [36] was used to measure psychosocial functioning. GAF-S consists of two subscales that score global psychosocial functioning and severity of symptoms, each scored between 1 and 100, representing low to high functioning last 7 days. These scores were based on information obtained through semistructured interviews with patients, conducted by blind raters. Raters had previously completed a web-based feedback training program for GAF-S scoring, shown to strengthen reliability and validity [37]. We also employed a procedure shown to further strengthen reliability [38] where relevant information from each interview was also conveyed to a second blind rater who gave an independent score, with the mean score of both raters determining the final score. The intraclass correlation between the independent raters was high (ICC 3.1 = .83, 95% CI: 0.78- 0.87) indicating satisfactory reliability.

### Secondary outcomes

*PTSD Symptom Scale - Self-Report (PSS-SR)* [30] consists of 17 items that assess PTSD symptoms along three symptom dimensions (reexperiences, avoidance, and hyperarousal). Items are scored based on frequency and severity of the symptom, on a Likert scale from 0 (not at all or only one time) to 3 (almost always or five or more times a week). PSS-SR has shown satisfactory psychometric properties, with a cut-off score of 14 that indicates clinically significant PTSD symptoms [39].

*Symptom Checklist 90 Revised (SCL-90 R)* [40] is a self-report measure of 90 items for psychological disturbances and distress. The Norwegian translation of SCL-90 R has shown good reliability and validity in clinical samples [41]. The summary scale Global Severity Index (GSI) was used as a measure of general psychopathology.

*Inventory of Interpersonal Problems (IIP-C)* [42] is a self-report measure to assess interpersonal difficulties with 64 items rated from 0 (not at all) to 4 (very much). The Norwegian version of IIP-C has shown satisfactory psychometric properties [43]. The mean score across all items was used as a general measure of interpersonal difficulties.

*Dissociative Experiences Scale-II (DES-II)* [44, 45] is a widely used and psychometrically well-validated 28-item self-report questionnaire that measures both pathological and non-pathological dissociative experiences. With an 11-point Likert scale, respondents indicate the percentage of their time dissociative experiences affect them. The overall mean of all items was used in this study as a score of dissociative symptoms.

All self-report-based measures were collected via a secure web-based platform (www.checkware.no) for ordinary use at the hospital. Instructions and access-code were provided to participants, and they could choose to submit their responses at the clinic or in private. Regular reminders were sent to participants that had not completed the measures.

### Statistical analyses

Demographics, clinical characteristics, and individual treatment- data were analyzed for group- differences at pre-treatment with t-tests for continuous variables and chi-square test for categorical data. Non-parametric tests were used if assumptions of normality were not met.

For outcome measures, we first calculated within-group pre-post effect-sizes based on Cohen’s *d* [46] using the formula $d=\frac{\mid {m}_1-{m}_2\mid }{\sqrt{S_1^2+{S}_2^2}-\left(2r{S}_1{S}_2\right).}$Pre-post scores for each condition were analyzed with paired sampled t-tests.

To test our prediction, that participation in group treatment combined with individual treatment would lead to greater improvements compared to individual treatment alone, differences in outcome trajectories between conditions were further analyzed with multilevel mixed-models (MLM) performed with the MIXED procedure in SPSS version 26. This analytical approach accommodates dependencies in nested observations, for instance in hierarchical data structures or longitudinal measurements of the same person [47]. To test the hypotheses three different MLMs were built:

The first model was built by testing a series of models for model fit, using Akaike Information Criteria (AIC) and − 2 log likelihood. The models were built by adding a fixed effect of intercept and time, random effects of intercept and time, and different covariance structures. When the best model was decided on, the treatment condition was added as a predictor, to test a condition x time interaction. The time variable was centered at the switching point, with negative values prior and positive values after the switching point. This model tested if the model implied intercept and the linear slope across all measurement points was different across the two treatment conditions.

The second model was a continuation of model 1 but was fitted with a linear-spline model with a knot at the switching point. This procedure breaks the time variable into two linear functions, allowing for condition x time interactions to be tested for different time-periods. The first linear function corresponds to the true experiment (see Fig. 2) and is a direct test of the effect of the group treatment versus the delayed treatment condition. The second linear function refers to the switching replication and is a test of the group treatment versus the follow-up condition. The intercept is a test of the difference across the conditions at the switching point.

The third model was also a continuation of the first model. This model retained one linear slope but had an interrupted timeline. That is, the timeline was recoded so that the intercept was moved to the end of the follow-up period for the EXP condition, whereas the intercept for the CTR condition remained at the switching point (i.e. at the end of the waiting list period). Accordingly, the estimation of the intercept is a test of the difference between the groups when the EXP condition had completed both the group therapy and subsequent individual therapy, whereas the CTR condition had not yet started the group therapy, but had received individual therapy from their individual therapists.

Statistical analyses were performed according to intent-to-treat principles. Missing data were handled using maximum likelihood estimation in the mixed models under the assumption of missing at random (MAR). In addition, we employed multiple imputations to obtain unbiased estimates of means, standard deviations, and effect sizes. Twenty datasets were generated for outcome measure values on all measurement points, with background variables and pre-treatment scores as predictors. Pooled estimates were used to calculate means, standard deviations, and effect sizes.

## Results

### Sample characteristics and threats to validity

Sample characteristics and differences between conditions are outlined in Tables 2 and 3. As expected based on previous studies, participants reported a high degree of childhood trauma, including sexual abuse. They also filled criteria for several other psychiatric conditions and the large majority reported previous inpatient treatment. Despite an average of 15 years since the first contact with mental health services, less than half of the participants reported previously being diagnosed with a dissociative disorder before participating in this study.

**Table 2:** Sample and group characteristics

Characteristic	Total (59)	EXP (29)	CTR (30)	Sample diff.
Demographics				
Age	35.2 (11.3)	34.7 (12.0)	35.6 (10.6)	*U* (59) = 480, *p* = .49
Female sex	93.2%	96.7%	89.7%	χ^2^ (59) = 1.15, *p* = .28
Married or partner	55.1%	59.1%	51.9%	χ^2^ (49) = 0.26, *p* = .61
College-level education	43.8%	50.0%	38.5%	χ^2^ (48) = 0.64, *p* = .42
Living with children	35.4%	40.9%	30.8%	χ^2^ (48) = 0.53, *p* = .46
Occupational status				
Work incapacity	81.3%	81.8%	80.8%	χ^2^ (48) = 0.01, *p* = .92
Student, full or part time	6.3%	9.1%	3.8%	χ^2^ (48) = 0.56, *p* = .45
Employed, full- or part-time	35.4%	36.4%	34.6%	χ^2^ (48) = 0.01, *p* = .90
Treatment history				
Years since first contact with mental health services	15.2 (10.7)	14.4 (10.2)	16.2 (11.5)	*U* (46) = 283, *p* = .55
Inpatient treatment ever	73.8%	77.8%	70.8%	χ^2^ (42) = 0.26, *p* = 0.61
Inpatient treatment last year	34.1%	27.8%	39.1%	χ^2^ (41) = 0.26, *p* = 0.61
Previous diagnosed dissociative disorder	43.5%	52.4%	36.0%	χ^2^(46) = 1.25, *p* = .26

*Note*: Data presented as means (SD) or percentages. *EXP* Experimental group, *CTR* Control group, *U* Mann-Whitney U statistic; χ^2^ = Chi Square Value

**Table 3:** Sample trauma-history and clinical comorbidity

	Total (59)	EXP (29)	CTR (30)	Diff. conditions
MINI Number of comorbid axis-I disorders	6.3 (2.6)	6.2(2.6)	6.5(2.7)	t (52) = 0.38, *p* = .71 (− 1.1 -1.7)
MINI any depressive disorder (present or lifetime)	90.7%	88.5%	92.9%	χ^2^ (54) = 0.31, *p* = .58
MINI any bipolar disorder (present or lifetime)	1.9%	0	3.6%	χ^2^ (54) = 0.95, *p* = .33
MINI severe suicidality (scored above 2)	33.3%	30.8%	35.7%	χ^2^ (54) = 0.15, *p* = .70
MINI any anxiety disorder (present or lifetime)	90.7%	92.3%	89.3%	χ^2^ (54) = 0.15, *p* = .70
MINI substance abuse	11.1%	15.4%	7.1%	χ^2^ (54) = 0.93, *p* = .34
MINI any psychotic disorder (present or lifetime)	33.3%	26.9%	39.3%	χ^2^ (54) = 0.93, *p* = .34
MINI any eating disorder	14.8%	11.5%	17.9%	χ^2^ (54) = 0.43, *p* = .51
MID total	41.5 (17.2)	39.5 (17.9)	43.2 (16.7)	U = 276, *p* = .28
CTQ total	76.1(19.0)	76.8(20.1)	75.4(18.3)	t (50) = − 0.26, *p* = .79 (− 8.6 - 11.4)
CTQ – Emotional abuse	94.2%	92.0%	96.3%	χ^2^ (52) = 0.44, *p* = .51
CTQ – Physical abuse	65.4%	64.0%	66.7%	χ^2^ (52) = 0.04, *p* = .84
CTQ – Sexual abuse	82.7%	80.0%	85.2%	χ^2^ (52) = 0.24, *p* = .62
CTQ – Emotional neglect	78.8%	84.0%	74.1%	χ^2^ (52) = 0.77, *p* = .38
CTQ – Physical neglect	84.6%	92.0%	77.8%	χ^2^ (52) = 2.02, *p* = .16
Number of CTQ abuse types	4.1 (1.2)	4.1 (1.1)	4.0 (1.2)	*U* (52) = 324, *p* = .80

*Notes*: Data presented as percentages or means (standard deviation). *EXP* Experimental group, *CTR* Control group, *U* Mann-Whitney U statistic; χ^2^ = Chi Square Value; *MID* Multidimensional Inventory of Dissociation, *CTQ* Childhood Trauma Questionnaire

We observed no statistically significant differences between the treatment conditions on background variables or outcome variables at baseline, indicating that the randomization procedure had produced similar samples. There were similarly no significant differences in frequency of individual sessions or therapeutic alliance between the two conditions.

### Attrition and missing data

As reported in Fig. 1, 14 patients (23%) dropped out during the study period. In both conditions, drop-out was higher in the period when patients received group treatment. The only significant difference between dropouts and completers on background and baseline variables was significantly higher mean MID scores in dropouts (50.9 vs. 35.8). Rates of missing data were highest for self-reported outcomes at the follow-up time points, with up to 41% missing values.

### Mixed models

**Fig. 3:**
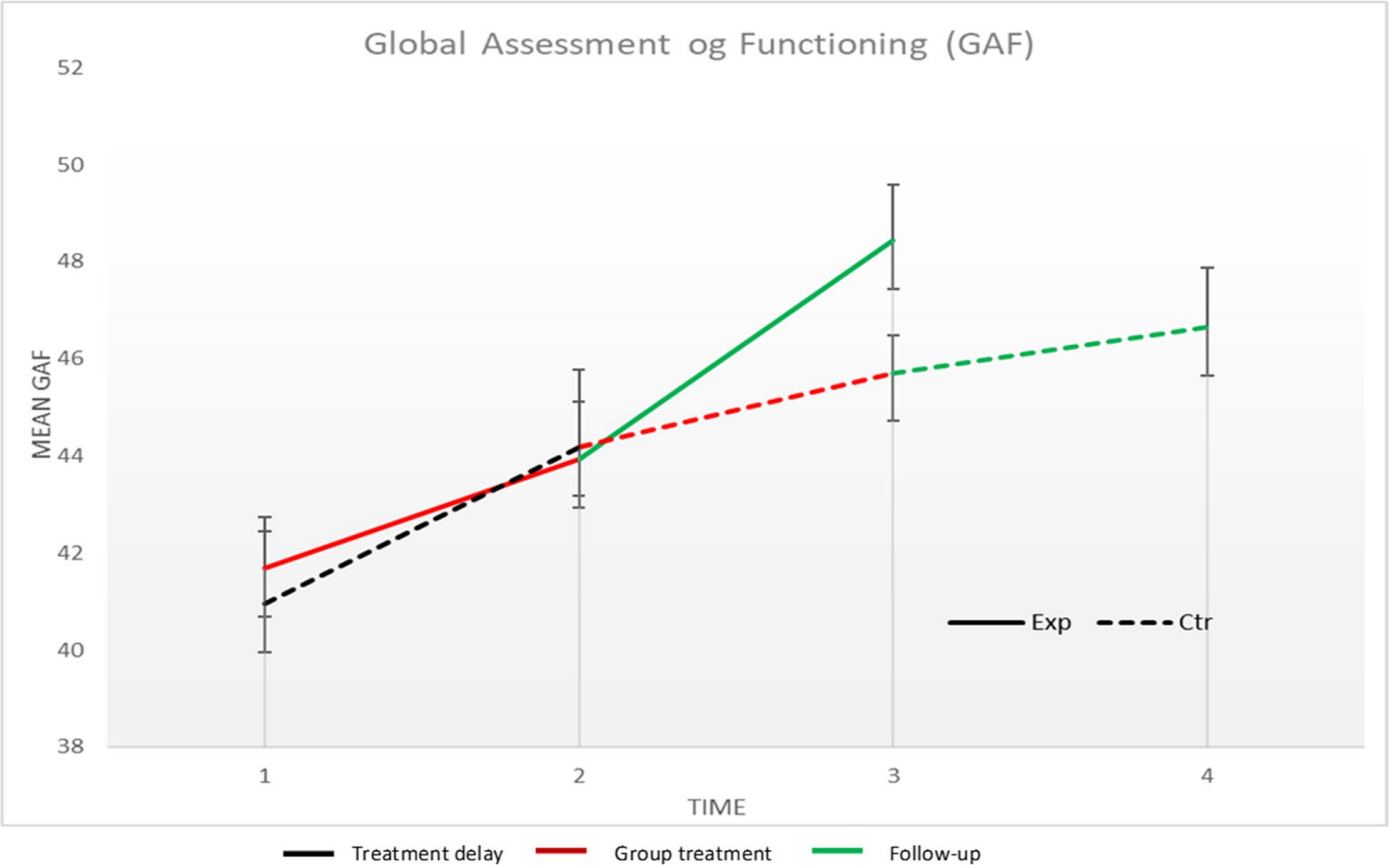
Global Assessment of Functioning across a randomized trial with delayed treatment control

Preliminary analysis: The model that provided the best model fit was estimated with a fixed effect of intercept and time, and random effects of intercept and time. Estimates for all models can be seen in Table 4. Trajectories of the main outcome across time-points can be seen in Fig. 3. Analyses using separate GAF scores from each assessor did not significantly affect outcomes or results.

**Table 4:** Multilevel mixed-models with GAF as dependent variable

Model		Parameters/Outcomes			
		Fixed parameters		Random parameters	
1	Linear	Intercept	42.6**(1.1)[40.5 – 44.7]	Intercept	19.5**(5.7)
	Model	Randomization	2.1 (1.5) [− 0.9 – 5.1]	Time	5.2**(3.3)
		Time	1.8* (0.7)[0.4 – 3.2]		
		Time *randomization	1.7 (1.1) [− 0.5 – 3.9]	Model fit	
				AIC	1008.1
				−2 Log RL	1000.1
			Fixed parameters	Random parameters	
2	Spline	Intercept	42.6** (1.3)[39.9 – 45.2]	Intercept	29.1**(8.8)
	Model	Randomization	1.1 (1.9)[−2.6 – 4.8]	Time1	52.0* (16.7)
		Time 1	1.8 (1.8)[−1.8 – 5.4]	Time 2	6.2 (4.5)
		Time 2	1.8* (0.8)[−0.1 – 3.4]		
		Time1 *randomization	0.2 (2.5) [−4.8 – 5.2]	Model fit	
		Time2 * randomization	3.5* (1.4)[0.7 – 6.3]	AIC	992.4
				−2 Log RL	978.4
			Fixed parameters	Random parameters	
2	Interrupted	Intercept	42.6** (1.1)[40.5 – 44.7]	Intercept	19.90**(6.5)
	time	Randomization	5.6* (1.7)[2.2 – 9.1]	Time	2.0 (2.4)
		Time	1.8* (0.6)[0.4 – 3.0]		
		Time *randomization	1.8 (1.0)[−0.3 – 3.8]	Model fit	
				AIC	1010.7
				−2 Log RL	1002.7

*Note*. Standard error in parentheses, 95% Confidence Intervals in brackets. *p < 0.05, **p < 0.01. *GAF* Global Assessment of Functioning

Model 1: The model estimated effect of intercept was significant (wiz. at the switching point, t = 40.5, *p* < .001), but the effect of condition on the intercept was not significant (t = 1.4, *p* = .16), indicating that the GAF-score at the intercept was significantly larger than zero, but not different between the conditions. The effect of time was significant (t = 2.6, *p* < .05), but the interaction between time and condition was not significant (t = 1.6, *p* = .12), meaning that the model estimated that the patients improved from the start of therapy to the end of follow-up, but that the two groups did not differ in their development over time (see also Table 4).

Model 2: The model estimated effect of intercept was significant (wiz. at the switching point, t = 32.6, *p* < .001), but the effect of condition on the intercept was not significant (t = 0.6, *p* = .56). Neither the first timeline (wiz. True experiment, t = 1.0, *p* = .32) nor the interaction between the first timeline and condition was significant (t = 0.01, *p* = .92). However, the second timeline was significant (wiz. The switching replication, t = 2.2, *p* < .05) as was the interaction between timeline and condition (t = 2.5, *p* < .05). That is, the model indicated that there was no significant change in GAF during the true experiment, and the conditions did not differ in their development. However, in the switching replication, there was both a significant change and a significant effect of condition where the EXP condition had a significantly larger change than the CTR condition.

Model 3: The model estimated effect of intercept was significant (wiz. at the end of follow-up for the EXP condition and the switching point for the CTR condition, t = 39.6, *p* < .001), and the effect of the condition on the intercept was significant, with the EXT condition having higher scores on GAF (t = 3.2, *p* < .01). That is, the model implies that when the EXP group has completed the follow-up period they have significantly higher scores on GAF than the CTR condition when they have completed the waiting period, with a large effect size (0.73). The timeline across all measurement points was also significant (t = 2.8, *p* < .01), but the interaction between time and condition was not significant (t = 1.7, *p* = .08).

### Outcomes

Outcome measures for each time point in both conditions can be seen in Table 4. We observed significant improvements in psychosocial functioning in both conditions across treatment, with medium to large effect-sizes. There was also a significant reduction in PTSD scores and general psychopathology in the control condition with medium effect-sizes, primarily related to symptom reductions during the follow-up phase. Other outcome measures did not show significant changes from pre-treatment to follow-up in either group (Table 5).

**Table 5:** Mean, Standard deviation, and effect sizes from assessment to follow-up

Measure and allocation	Assessment	T1 Pre treatment	T2 Switching - point	T3 Switching replication	T4 Follow up CTR	Effect size (*d*) Pre – switching point	Effect size (*d*) pre – follow up
GAF							
EXP		41.7 (5.7)	43.9 (6.3)	48.4 (6.3)		0.4 (−0.1 – 0.9)	1.1 (0.6 – 1.7)**
CTR		40.9 (8.2)	44.2 (8.7)	45.7 (4.2)	46.6 (6.7)	0.4 (−0.1- 0.8)	0.7 (0.3-1.2)**
PSS-SR							
EXP	32.9 (6.3)	33.3 (5.5)	33.2(6.5)	33.3 (6.9)		0.0 (−0.5 – 0.4)	−0.1 (− 0.6 – 0.4)
CTR	34.6 (8.3)	34.6 (7.3)	36.5 (6.4)	34.5 (5.9)	29.5 (7.9)	−0.2 (−.0.6 – 0.1)	0.6 (0.1 – 1.1)**
DES							
EXP	34.6 (29.8)	36.5 (15.9)	38.2 (16.1)	36.0 (16.6)		−0.1 (−0.5 – 0.2)	−0.1 (− 0.4 – 0.3)
CTR	41.9 (21.2)	47.8 (18.6)	45.4 (13.6)	40.8 (13.6)	37.9 (11.5)	−0.1 (− 0.6 – 0.3)	0.2 (− 0.3 – 0.7)
SCL90							
EXP	1.8 (0.9)	1.8 (0.7)	1.7 (0.8)	1.7 (0.9)		0.1 (−0.4 – 0.6)	0.1 (−0.4-0.6)
CTR	1.9 (0.7)	1.9 (0.9)	1.9 (0.9)	1.9 (1.0)	1.6 (0.8)	0.0 (−0.4 – 0.4)	0.4 (−0.1 – 0.9)*
IIP							
EXP	1.7 (0.6)	1.8 (0.1)	1.7 (0.6)	1.7 (0.6)		0.0 (−0.6 – 0.5)	0.0 (−0.4 – 0.4)
CTR	1.7 (0.5)	1.7 (0.7)	1.7 (0.5)	1.6 (0.8)	1.4 (0.7)	0.0 (−0.3 - 0.3)	0.4 (0.0 – 0.9)

*Note*: Data displayed as mean (std. deviation) or effect size (95% confidence interval). *p < 0.05, **p < 0.01, based on paired-samples tests; *GAF* Global Assessment of Functioning, *PSS-SR* PTSD Symptom Scale Self Report, *DES* Dissociative Experiences Scale, *IIP 64* Inventory of Interpersonal Problems 64, *SCL* 90 = Symptom Checklist 90

## Discussion

The aim of this study was to evaluate the treatment of patients with CDD that combined individual treatment with psychoeducational group-based stabilization – treatment. We did not observe differences in treatment trajectories consistent with our prediction that group treatment would lead to greater improvements than individual treatment alone since trajectories were parallel before the primary outcome assessment. This indicates that the studied stabilization treatment does not immediately produce superior outcomes compared to individual treatment alone. We observed overall improvements in psychosocial functioning, as measured with GAF, with large pre-post effect sizes, in both conditions. However, this effect was only significant over the 6-month follow-up period. Self-reported symptoms behavior remained mostly unchanged, with only a small decrease in PTSD and general psychiatric symptoms in the control condition being significant.

We did however find indications that the effects of group treatment on psychosocial functioning were more profound in the follow-up period, with differences in intercepts indicating a between-group effect of medium size. Similarly, we found that the significant improvements in self-reported symptoms in the control condition occurred in the period following group-participation. These findings should be interpreted with caution, as our delayed-treatment design did not allow for a direct comparison of follow-up effects and the control condition but may point to a possible positive long-term effect of the study-treatment. This is in line with the rationale for stabilizing treatment for dissociative disorders. Boon and colleagues [25] describe that the stabilizing groups “( …) *highlights ways for both the dissociative patient and the therapist to effectively work with an underlying dissociative organization of the personality as an essential part of coping with many of the well-known symptoms of chronic traumatization*”. ISSTD guidelines [12] also describe skills training as an essential part of stabilization. Understandably, the effect of newly acquired skills and improved mutual understanding of dissociative processes for patients and therapists will be more prolonged rather than immediate. Clinical experience indicates that many patients require a longer time to process the material and learn to manage dissociation and other symptoms. It is also important to underline that the study treatment was an abbreviated 20 session version of the 43 session treatment outlined by the treatment manual [25] so a longer intervention might have improved outcomes.

The observed improvements in psychosocial functioning suggest that participants in the current study improved as expected in the first stabilization - phase of treatment. An earlier naturalistic study [19] can serve as a comparison since GAF scores of CDD patients were reported from cohorts in different phases of treatment, as defined by ISSTD guidelines. The scores of the phase 1 cohort (M = 44.7) were higher than pre-treatment GAF scores in the current study (M = 41.3), while the phase 2 cohort scores (M = 48.7) were comparable to the post-treatment scores (M = 47.5). Accordingly, patients in our study had poorer initial psychosocial functioning but were brought significantly towards the next phase of treatment more quickly than in the naturalistic sample, where patients in the second phase had been in treatment for a mean of 4.1 years [19].

As with previous empirical investigations [17, 48], the results from the current study do not support the hypothesis that treatment of CDD is associated with iatrogenic harm. According to some scholars [13–15], interventions focused on dissociative parts will reinforce the patient’s incorrect beliefs about having multiple identities, thereby worsening symptoms and functioning and causing harm to patients. For the first time in a randomized controlled design, we show that treatment following expert guidelines [12] rather is associated with improvements in psychosocial function and not significant deterioration or harm.

However, it is important to acknowledge that the clinical effects of this study on self-reported symptoms were either small or insignificant. Despite improvements in psychosocial functioning, most of the participants still experienced high degrees of suffering and disturbances. Most noteworthy, little changes in dissociative symptoms as measured with DES occurred, indicating that the treatment failed to facilitate change in the core features of the disorders. A recent study using an online intervention together with regular individual therapy, observed larger effects on dissociation scores over a 2-years period, underscoring that longer treatment might be necessary to affect these symptoms [24]. Also, the online intervention had less initial focus on dissociative parts and inner cooperation than the treatment of the present study. Patients with CDDs might need a more gradual emphasis on dissociative parts to avoid being overwhelmed and facilitate change. Furthermore, stabilization treatment may be more effective when delivered in an individual format more easily adapted to each patient’s needs.

However, the phase-based model that forms the basis of ISSTD guidelines [12] and the study-treatment has been challenged recently, both as it applies to the treatment of PTSD [49] and CDD [50]. Critics argue that processing of trauma without a preceding stabilization-phase may be more effective [51]. Evidence-based treatments for other psychiatric disorders have also been suggested as potentially effective for CDD [51, 52]. Our results highlight that there is much room for improvement in the treatment of patients suffering from CDD, and the importance of continued innovation in interventions and treatment delivery. We can not however conclude if a stabilization phase is necessary or not, and further research should investigate both phase-based and trauma-focused interventions. Together with methodologically sound clinical research, this may improve options for patients and clinicians. In addition, qualitative research can inform how patients with CDD experience treatment and what outcomes they view as most important for them [53]. Qualitative interviews have been conducted with a subsample of this trial that will be analyzed and presented in the future.

Several limitations to our study are important when interpreting the results. First, the sample of the study is small and we very not able to recruit as many participants as originally planned. Together with attrition, this reduced the statistical power to detect statistically significant effects and thereby increased our type II error rate. Also, we experienced a large proportion of missing data, especially in self-reported measures, which weakens the confidence of our estimates. We did not control or protocol individual therapies. Although we collected data on content and frequency, other possible confounding variables might have affected differences between conditions. For instance, individual therapists might have also offered stabilization-focused interventions. We did not record or control for how much the patients used the skills or videos from the group treatment, or to what degree these were incorporated in the individual therapies. As previously mentioned, the delayed treatment design that was employed made it difficult to infer differences after the follow-up period. The group assessments and group interventions were conducted by well-trained and experienced clinicians, but we did not test interrater reliability or collect observer-based data on fidelity. Our outcome measures may have been less sensitive to change in CDD patients than measures specifically designed for this patient group [54]. And finally, we did not control for medication use. However, this study is to our knowledge the first clinical study of the treatment of CDD that includes a randomized design and a control condition. Other strengths are that the inclusion of participants was based on ordinary clinical referrals and transparent criteria of inclusion and exclusion, strengthening the generalizability of our results. We employed robust statistical methods testing different linear mixed-models. Since the study treatment was protocolled and based on a published treatment - manual, the results can more easily influence clinical practice and make replication studies possible.

Our results show that participation in stabilizing group treatment does not lead to superior outcomes for patients with complex dissociative disorders. However, we show that psychological treatment of complex dissociative disorders is associated with improvements in psychological functioning, but only at a 6-months follow-up. This study is the first randomized controlled trial for the treatment of complex dissociative disorders and there is an urgent need for more clinical research on treatment for this patient group.

## Supplementary Information

**Additional file 1.**

## Data Availability

The datasets generated and/or analyzed during the current study are not publicly available due to ethical approval and confidentiality agreements made with participants, but are available from the corresponding author on reasonable request.
