# Supplementary material for: Group treatment for complex dissociative disorders: a randomized clinical trial
Source: BMC Psychiatry. 2022 May 16;22:338. doi: 10.1186/s12888-022-03970-8 (PMC9112598; doi:10.1186/s12888-022-03970-8)
Supplement: Supplementary file 1 — Additional file 1. [file 12888_2022_3970_MOESM1_ESM.docx]

**Supplementary 1**

**Characteristics of conventional individual therapies**

At each time-point, patients and individual therapists were asked to fill out a generic form asking about therapist background, characteristics of the therapy (length, frequency, working alliance) and to what degree the individual treatment was adapted in the period the patient attended stabilization group.

**Characteristics of individual therapists**

| Gender | 85,4 % Female |
| --- | --- |
| Age | 20-30 years:  31-40 years: 39%  41-50 years: 39 %  51-60 years: 17,1 %  60 + years: 4,9% |
| Professional background | Doctor / Psychiatrist: 20,5%  Clinical Psychologist: 74,4%  Nurse: 2,6%  Physical therapist: 2,6% |
| Work setting | Mental Health Clinic: 63,4%  Private practice: 29,3%  Inpatient hospital: 7,3% |
| Experience as therapist | 0-5 years: 23,7%  6-10 years: 23,7%  11-15 years: 18,4%  16-20 years: 15,8%  21-30 years: 13,2%  31+ years: 5,3% |
| Training in trauma-treatment* | 70% |
| Training in trauma-focused treatment** | 37,5% |
| Previous experience as individual therapist for patient participating in stabilization group | 32,5% |
| *Respondents indicated attending one or more courses or workshops about trauma-treatment  ** Respondents indicated attending one or more courses or workshops about trauma-focused therapy, such as EMDR, Trauma focused CBT, Prolonged Exposure or Narrative Exposure Therapy | |

**Characteristics of individual therapies**

| Reported length of individual treatment at T1 | < 1 month: 7,5%  1 – 3 months: 7,5%  3-6 months: 15%  6 – 12 months: 17,5%  1 – 2 years: 22,5%  2-4 years: 17,5%  4+ years: 12,5% |
| --- | --- |
| Number of individual sessions during participation in group therapy | < 5: 10%  5-10: 10%  11-15: 30%  16-20: 15%  21-25: 17,5%  26-30: 5%  >30%: 12,5% |
| Number of individual sessions during wait period (CTR) | < 5: 13%  5-10: 13%  11-15: 8,7%  16-20: 8,7%  21-25: 21,7%  26-30: 13%  >30: 21,7% |
| Working Alliance Inventory (WAI) at T1 | Therapist rated: Mean 4,7, SD 0,7  Patient rated: Mean 4,8, SD 1,3 |

**Patient-reported adaptations of individual therapy in period corresponding to patient attending group treatment**

| I have talked about experiences from group treatment | Never: 0%  Sometimes: 47,5%  Often: 37,5%  Very often/always: 15% |
| --- | --- |
| My therapist has asked about experiences from group treatment | Never: 5%  Sometimes: 55%  Often: 25%  Very often/always: 15% |
| The topic of the psychoeducation in group has been a topic in the individual therapy | Never: 10%  Sometimes: 50%  Often: 25%  Very often/always: 15% |
| We have reviewed the psychoeducation from group in the individual sessions | Never: 25,6%  Sometimes: 43,6%  Often: 20,5%  Very often/always: 10,3% |
| My therapist has helped me with homework from group | Never: 53,8%  Sometimes: 30,8%  Often: 10,3%  Very often/always: 5,1% |
| Exercises from group have been practiced in individual sessions | Never: 43,6%  Sometimes: 38,5%  Often: 12,8%  Very often/always: 5,1% |

**Supplementary 2**

**Deviations from protocol**

*Sample size:* The protocol originally planned to include 72 participants (36 in each condition), based on a priori power analysis. However, due to a lower prevalence of CDD in the study population than predicted and resource constraints, the final sample recruited was 59 participants.

*Treatment:*  There were minor changes to the experimental group program, most notably that the topic “learning to regulate your self” was moved to the start of the program. The rationale was to learn more self-regulation before addressing dissociation in the next session.

*Outcome measures:*

- The HRV-data collected has not yet been analyzed and will be published in a separate paper.
- SF-36 was not used due to licensing issues with the hospital making it difficult to obtain the correct syntax for scoring
- The data from Behavior Checklist has not been reported because of very high rates of missing data and skewed distributions on the items. It is therefore difficult to analyze and obtain reliable estimates.
